# Flexible Thermoelectric Type Temperature Sensors Based on Graphene Fibers

**DOI:** 10.3390/mi14101853

**Published:** 2023-09-28

**Authors:** Chenying Wang, Yuxin Zhang, Feng Han, Zhuangde Jiang

**Affiliations:** 1 School of Instrument Science and Technology, Xi’an Jiaotong University, Xi’an 710049, China; wangchenying@mail.xjtu.edu.cn; 2 State Key Laboratory for Manufacturing Systems Engineering, Xi’an Jiaotong University, Xi’an 710049, China; zdjiang@xjtu.edu.cn; 3 International Joint Laboratory for Micro/Nano Manufacturing and Measurement Technologies, Xi’an Jiaotong University, Xi’an 710049, China; 4 The 41st Institute of the Fourth Academy of CASC, Xi’an 710049, China; yxz_2023@163.com

**Keywords:** graphene fibers, vitamin C, thermoelectric, flexible temperature sensors

## Abstract

Graphene, as a novel thermoelectric (TE) material, has received growing attention because of its unique microstructure and excellent thermoelectric properties. In this paper, graphene fibers (GFs) are synthesized by a facile microfluidic spinning technique using a green reducing agent (vitamin C). The GFs have the merits of high electrical conductivity (2448 S/m), high flexibility, and light weight. Further, a flexible temperature sensor based on GF and platinum (Pt) with a sensitivity of 29.9 μV/°C is proposed, and the thermal voltage output of the sensor can reach 3.45 mV at a temperature gradient of 120 °C. The sensor has good scalability in length, and its sensitivity can increase with the number of p-n thermocouples. It has good cyclic stability, repeatability, resistance to bending interference, and stability, showing great promise for applications in real-time detection of human body temperature.

## 1. Introduction

Changes in human body temperature are one of the most important indicators of human activity and health, helping to maintain the thermal balance between the body and its surroundings, while some physical sensations related to emotions can also be observed by analyzing subtle changes in body temperature. It is also known that changes in human body temperature can be used as a primary medical diagnostic assessment indicator for new crown pneumonia. Flexible wearable temperature sensors provide a method for real-time monitoring of human body temperature, which can be classified into thermal resistance, thermocouple, and fiber optic types according to the temperature measurement principle [1,2,3]. Among them, thermocouple temperature sensors are usually made of two materials. Based on the thermoelectric principle of temperature measurement, the temperature signal is converted into a voltage signal for output, and the resulting thermoelectric potential has a linear or near-linear relationship with the temperature difference [4]. Traditional thermocouple temperature sensors usually use metal as the thermal electrode, which is rigid and difficult to bend and cannot accurately measure the temperature on complex curved surfaces [1]. In addition, the high cost of precious metal materials limits the further development of thermocouple temperature sensors. With the increasing demand for comfort in wearable devices, flexible thermocouple temperature sensors, which are light in quality and highly flexible, are gradually gaining popularity. Benefiting from its flexibility and adaptability, it can be directly attached to the human skin to record the temperature of the human body and the external environment for a long time. Thermoelectric materials are one of the most important components in thermocouple temperature sensor research. Compared with traditional thermoelectric materials, flexible thermoelectric materials are bendable in shape, small in size, and light in mass and have good application prospects in wearable devices and other flexible electronics. Graphene is a thermoelectric material with excellent performance that has the advantages of non-toxicity, abundant raw materials, high conductivity, high electron mobility and carrier concentration, etc. [5,6,7]. Dragoman et al. proposed a graphene-based metal electrode device, according to which the transfer matrix method was used to predict the Seebeck coefficients up to 30 mV/K [8].

Graphene fiber (GF) is a type of macrofiber material that consists of individual units of graphene and its derivatives. There have been numerous methods employed to directly construct macrofibers using graphene sheets [9,10]. This material harnesses the remarkable characteristics of a single graphene sheet, including its exceptional mechanical strength, electrical conductivity, and thermal conductivity. Not only does it have mechanical flexibility and knittability similar to that of conventional fibers, but it is also lower cost, lighter, and more malleable than conventional carbon nanotube fibers, while it is easier to functionalize it in situ or in post-synthesis, which is of significant practical application value. However, the graphene fiber-based flexible thermocouples obtained from the current study still suffer from the problem of low sensitivity.

In this paper, graphene oxide fibers (GOFs) were proposed utilizing a microfluidic spinning technique, and graphene fibers were synthesized using vitamin C as a reducing agent. The impact of various concentrations of vitamin C in the graphene oxide (GO) solution on the electrical conductivity and thermoelectric properties of GF was examined. A lightweight, highly flexible, and inflexible thermocouple temperature sensor was designed using GF and platinum (Pt) electrodes and calibrated and tested with a sensitivity of up to 29.9 μV/°C. The sensor has good scalability in terms of length and can be made more sensitive as the number of thermocouple pairs comprising it increases.

## 2. Experimental Section

### 2.1. Materials and Reagents

The modified Hummer’s method [11] was used to prepare the GO. Vitamin C was purchased from Sigma-Aldrich Co., Ltd., St. Louis, MO, USA.

### 2.2. Preparation of Graphene Fibers

**Figure 1:**
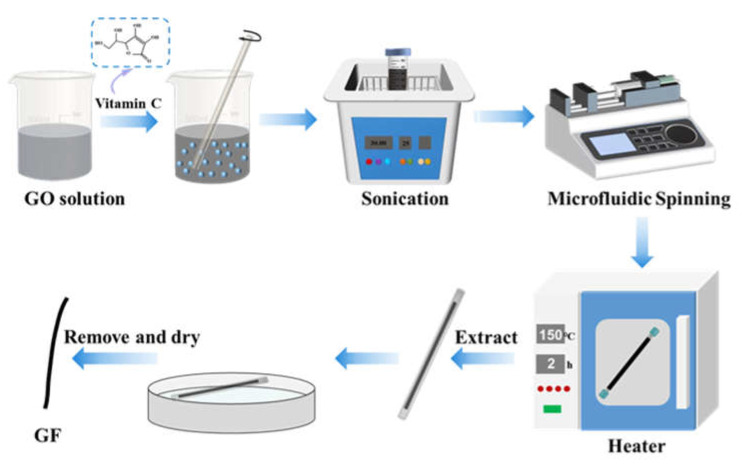
Schematic diagram of preparation process of GFs.

An approach to fabricating graphene fibers using a facile microfluidic spinning method is proposed, as illustrated in Figure 1. Firstly, a well-dispersed 12 mg/mL graphene oxide solution was prepared after ultrasonication for 30 min. Vitamin C was added to the GO solution at different concentrations of 0 wt%, 1 wt%, 2 wt%, 3 wt%, and 4 wt%. These concentrations were labeled as GOFs, 1 wt%-GFs, 2 wt%-GFs, 3 wt%-GFs, and 4 wt%-GFs, respectively. The mixture was then thoroughly mixed to ensure homogeneity. Secondly, the graphene oxide solution was slowly injected into polytetrafluoroethylene (PTFE) tubes with a 0.5 mm inner diameter. The PTFE tube was put into an oven and subjected to a temperature of 150 °C for a duration of 2 h. While the water steadily evaporated, fibers began to develop within the tube. Subsequently, GOFs and GFs matching the geometry of the tube were pushed out of the tube with the help of N2 flow and dried in an atmospheric environment.

### 2.3. Fabrication of TE Temperature Sensors

**Figure 2:**
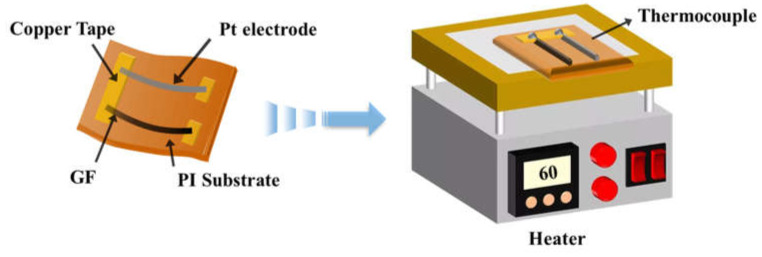
GF/Pt thermocouple preparation diagram.

As exhibited in Figure 2, the flexible temperature sensor was formed by combining two materials, with GF as the anode and the platinum (Pt) electrode as the cathode. The GF (about 2 cm long) and the Pt (about 2 cm long) were connected on a copper foil tape through the conductive silver paste and then mounted on a polyimide (PI) substrate (40 mm long × 20 mm wide). The connecting end of the two electrodes was the hot end of the thermoelectric-type temperature sensor, and the other ends of the anode and cathode electrodes were both the cold ends of the sensor. During the heating process, the hot end of the senor was put on the hot plate and heated by the silicone rubber heating belt as the heat source, while the cold end was placed in the air at 25 °C. The temperature of the hot plate can be controlled and adjusted by an intelligent temperature control system. The TE temperature sensor was obtained by applying a temperature difference between the hot and cold ends to measure the output voltage.

### 2.4. Characterization and Measurements

A field emission scanning electron microscope (FESEM, SU-8010, Hitachi, Tokyo, Japan) was employed to capture morphological images of fibers. And a laser Raman spectrometer (HR800, Horiba Jobin Yvon, Paris, France) was applied to get the Raman spectroscopy. The chemical composition and electronic structure of the sample surface were analyzed using a Thermo Fisher Escalab xi + X-ray photoelectron spectroscopy analyzer (XPS, ESCALAB Xi+, Thermo Fisher, Waltham, MA, USA). Conductivities were measured using Source Meter (2450, Keithley, Beaverton, OR, USA). The output voltage was measured using a data collector (LR8450, HIOKI, Nagano, Japan). A thermoelectric performance test system was established that can calibrate and test the sensor in a wide low-temperature range. The hot end of the thermocouple was heated by the silicone rubber heating belt as the heat source, and its temperature was controlled and adjusted by an intelligent temperature control system. Additionally, the data collection device was utilized to measure the temperature at both the hot and cold ends, which were monitored using a standard K-type thermocouple. It also recorded the output voltage of the developed thermocouple.

The sensitivity (Seebeck coefficient) of a thermocouple temperature sensor is calculated using the following formula [12]:

(1) $$S=\frac{dV}{d\Delta T}=\frac{dV}{d(T_{h}-T_{c})}$$

*V*—Output thermopotential of the sensor; *T_h_*—Temperature of the hot end; *Tc*—Temperature of the cold end; *S*—Sensitivity of the thermocouple.

## 3. Results and Discussion

**Figure 3:**
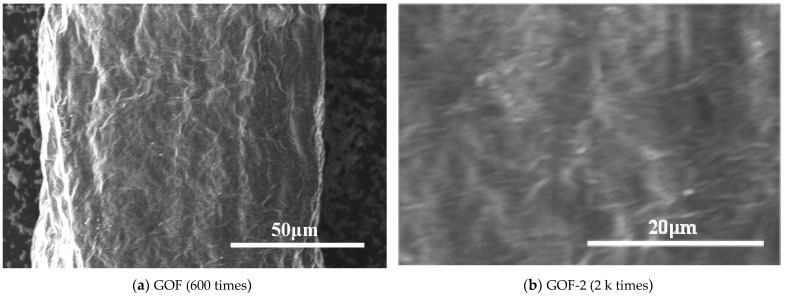

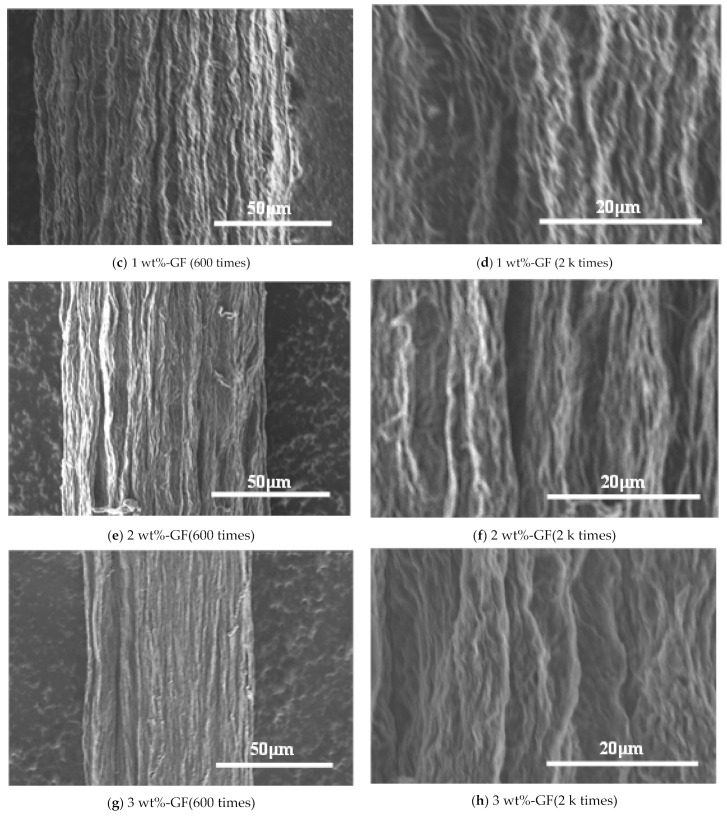

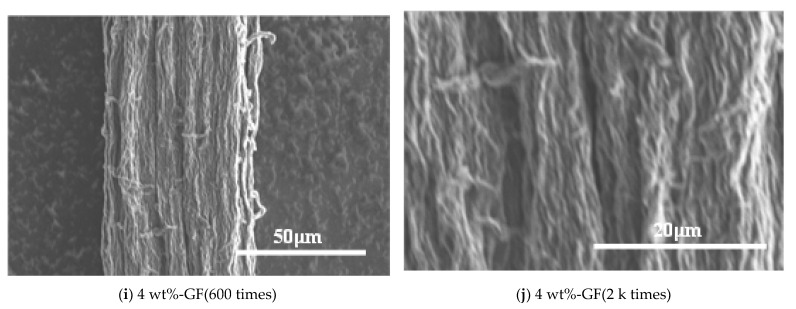
SEM images of the GOFs and GFs with different vitamin C doping content.

The surface morphologies of the fibers with varying levels of vitamin C were investigated by SEM, as depicted in Figure 3. Figure 3a,b show the surface morphologies of GOF with a diameter of about 110 μm captured at different magnifications of 600 and 2 k, respectively. Compared with GOF, the diameter of GF is significantly reduced. GOF has a paper-like and less wrinkled surface with a disordered, smooth-surfaced fluffy appearance, whereas GF shows significant curvature and more wrinkles. The diameter of the fibers decreases gradually with the increase in vitamin C doping. The surface morphology of GF with different amounts of vitamin C doping shows no significant difference, and all show obvious bending and folded structures. The presence of van der Waals forces and the strong π-π stacking between graphene sheets cause GO sheets to easily clump together. However, the introduction of vitamin C prevents this clumping. Meanwhile, GO has abundant oxygen functional groups, including carboxyl (COOH), carbonyl (-C=O), epoxide (C-O-C), and hydroxyl (-OH) groups, whereas vitamin C can remove a large number of oxygen groups within GO during the chemical reduction process, leading to the appearance of wrinkles after the reduction process [13,14,15].

**Figure 4:**
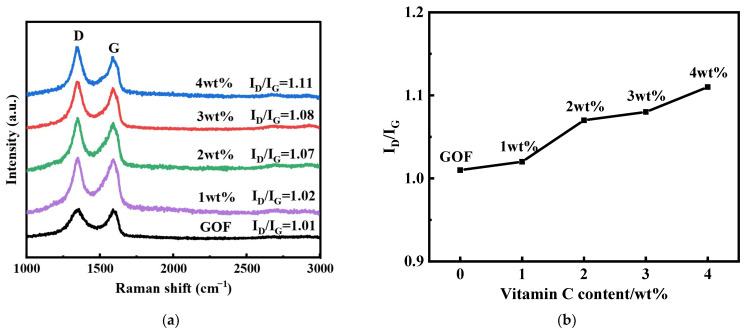
Raman spectra of GFs with different amounts of vitamin C doping. (**a**) Raman spectra of GOFs and GFs; (**b**) Comparison of Raman results between GOFs and GFs.

The Raman spectra of GOFs and GFs with different C doping are illustrated in Figure 4. Raman spectra are widely used for the structural characterization of graphene and related materials. There are two distinct peaks observed in the spectrum. The first peak, known as the D peak, appears at 1350 cm^−1^ and is attributed to the telescopic vibration of the sp^3^ carbon atom. This peak is indicative of structural deficiencies and disorders. The second peak, called the G peak, is located at 1590 cm^−1^ and is associated with the E_2g_ vibrational mode of the sp^2^ carbon domain [16,17]. The degree of defects and disorder in graphene structures can usually be characterized by the ratio of relative intensities of the D and G peaks (defined as I_D_/I_G_). After vitamin C reduction, the I_D_/I_G_ values of 1 wt%-GF, 2 wt%-GF, 3 wt%-GF, and 4 wt%-GF are 1.02, 1.07, 1.08, and 1.11, respectively, which all increase compared with that of GOF (1.01). The increased I_D_/I_G_ values indicate that the oxygen-containing functional groups were effectively removed during the chemical reduction process, leading to the introduction of more structural defects. Carbon-carbon bonds on the surface of the graphene structural layer are broken, and after reduction, the new forming sp^2^ domains are smaller in size but more ubiquitous.

**Figure 5:**
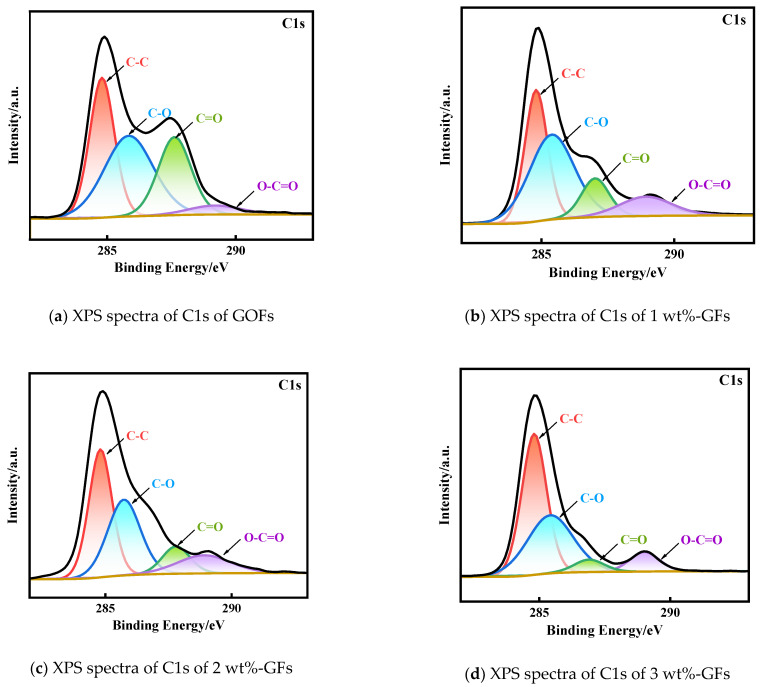

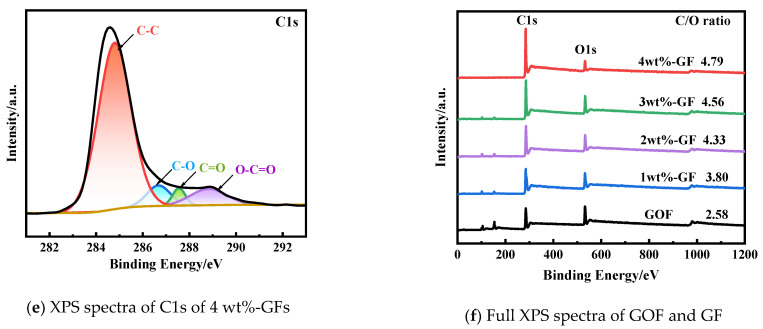
XPS spectra of GFs with varying levels of vitamin C.

The XPS results of GOF and GF are presented in Figure 5. Analysis of Figure 5f reveals the presence of C1s (284 eV) and O1s (532 eV) based on the observed peaks. By performing deconvolution on the high-resolution C1s peak of GOF, it is observed that there are four peaks corresponding to C-C, C-O, C=O, and O-C=O, centered at 284.8 eV, 285.4 eV, 286.6 eV, and 288.7 eV, respectively. Upon annealing, GF exhibits a similar and significant decrease in these peaks, indicating the partial decomposition of oxygen functional groups and the partial recovery of π-conjugated structures after the addition of vitamin C. The profiles of GF with different levels of vitamin C were approximately the same. The differences in the C/O ratios were evident in the XPS full spectra, which were 2.58 for GOF, 3.80 for 1 wt%-GF, 4.33 for 2 wt%-GF, 4.56 for 3 wt%-GF, and 4.79 for 4 wt%-GF. After vitamin C reduction, the C/O ratios all increased, indicating the effectiveness of vitamin C on GOF deoxygenation. The largest C/O ratio was found for 4 wt%-GF, proving that the highest degree of fiber reduction was achieved with the addition of 4 wt% vitamin C.

**Figure 6:**
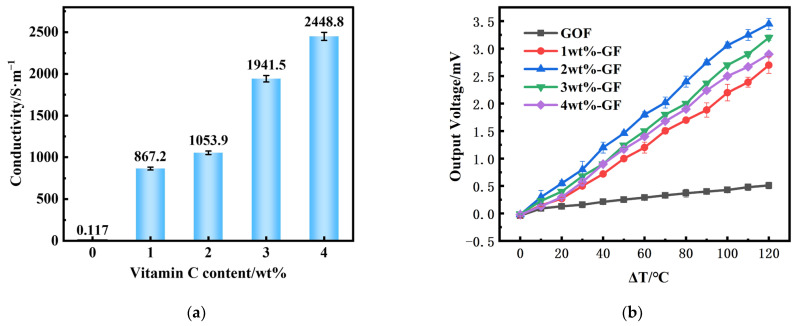
Electrical and thermoelectric properties of GFs. (**a**) Electrical conductivities of GFs at different vitamin C doping levels; (**b**) Thermoelectric output curve of GF/Pt thermocouple.

The effect of vitamin C on the degree of reduction of graphene fibers was analyzed in Figure 6a. The electrical conductivity of GOF is very poor (0.117 S/m), and that of GF rises rapidly after the addition of vitamin C. Through the increased vitamin C content, the electrical conductivities of GFs increase rapidly. The phenomenon occurs because the oxygen functional groups, including -OH, C-O-C, and -COOH, start to break down when vitamin C is reduced. This leads to the deoxygenation of sp^3^ hybridized carbon atoms, allowing them to regain their sp^2^ hybridized state and restore the conjugated C=C bond and π bond over time. Additionally, when the fiber is heat treated at 150 °C, the decomposition of oxygen-containing functional groups causes a gradual decrease in resistance and a rapid increase in conductivity. The 4 wt%-GF has the largest conductivity of 2448.8 S/m. Combined with the characterization analysis in the previous section, the trend of the conductivity is the same as that of the C/O ratio in the XPS analysis. The conductivity of graphene fiber is a direct criterion to judge its reduction effect, and in summary, it can be concluded that the treatment with vitamin C can significantly improve the conductivity of GFs and successfully reduce GOFs to obtain GFs.

To investigate the thermoelectric characteristics of graphene fibers, a platinum wire was used as a reference electrode with GF at different vitamin C doping levels to form a thermocouple to test its thermoelectric properties. Since the Seebeck coefficient of a standard Pt wire is very small, about 1.67 µV/°C [18], Pt can be used as one of the hot electrodes in the thermocouple, and GF at different annealing temperatures can be used as the other hot electrode. The output voltage characteristics of the thermocouple can be examined to qualitatively compare the influence of vitamin C doping on the thermoelectric performance of GF. An autonomous thermoelectric test system was used to test the performance of GOF/Pt and GF/Pt thermocouples, and the output voltages were surveyed by means of a temperature difference applied at both ends of the thermocouples under different steady-state temperature differences. The cold end of the thermocouple was placed in the air at about 25 °C. The output thermopotential of the thermocouple was recorded during the warming process as the temperature of the heating tape was regulated to increase the hot end temperature. The correlation between the output voltage of the thermocouple and the temperature difference between the two ends can be observed in Figure 6b. The thermopotential in the graph shows good linearity in the range of temperature differences of 0–120 °C. It can be concluded that the Seebeck coefficient of the material undergoes a significant enhancement after the reduction with vitamin C. The material has a very good thermal potential. The maximum output voltage of the thermocouple (3.45 mV) was observed at the introduction of 2 wt% of vitamin C. The output voltage of the thermocouple was also found to be higher than that of the original material. Due to the presence of oxygen impurities, the pristine graphene fiber exhibits p-type majority carriers and is a p-type thermoelectric material. As can be seen in Figure 6a, the electrical conductivity of GOF rises after the addition of vitamin C. This is because the oxygen functional groups in GF are reduced with the increasing addition of vitamin C. However, the output voltage of the thermocouple has not shown the same trend with the increase in vitamin C (shown in Figure 6b). According to Formula (1), the output voltage is determined by the Seebeck coefficient of materials. The Seebeck coefficient of GF is affected not only by conductivity but also by the Fermi level. As indicated in the Raman characterization (Figure 4a), more structural defects are introduced with the increasing content of vitamin C treatment. When the vitamin content increases from 0 to 2%, the Fermi level state of graphene is improved, resulting in a further increase in the Seebeck coefficient. When the vitamin content increases from 2 to 4%, excessive structural defects will affect the Fermi level state of graphene, leading to a decrease in the Seebeck coefficient. Thus, the output voltage will vary with changes in the Seebeck coefficient [19].

It is important to note that the measured thermal voltage output is influenced not only by the temperature difference between the hot and cold junctions but also by the Seebeck coefficient of the thermoelectric material. To further characterize the relation between thermal voltages and temperature differences, the results of fitting the thermoelectric output curves of the thermocouple temperature sensors with different vitamin C contents (fit R2 > 0.97) are shown in Table 1. The Seebeck coefficient of 2 wt%-GF/Pt was the largest (29.9 μV/°C). Vitamin C has the ability to eliminate the oxygen-containing functional groups present in GO. This process enhances the movement of charge carriers within the graphene layer, resulting in improved carrier mobility. Consequently, the Seebeck coefficient, which measures the conversion of temperature difference into electrical voltage, is enhanced.

It has been proven that the use of green and harmless reducing agents, such as tea extract, ethylene glycol, sugar, and vitamin C, is an environmentally friendly, mild, and effective method to obtain reduced graphene oxide (RGO) [20]. Vitamin C, also known as ascorbic acid [21], has the advantages of low cost, non-toxicity, and environmental friendliness.

**Figure 7:**
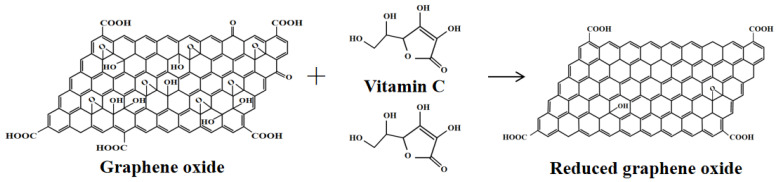
Mechanistic analysis diagram of chemical reduction of GO.

The chemical reduction mechanism of GO is shown in Figure 7. The essence of GO chemical reduction is the reaction between the reducing agent and one or more functional groups on GO. Due to the different reactivity of each oxygen-containing functional group to GO, different reducing agents have different effects on removing functional groups. Moreover, vitamin C is rich in flavonoids, which react with the GO solution and undergo oxidation by the oxygen groups on the surface of the GO sheet. This reaction leads to the formation of benzoquinone products. Hydrogen ions can act as a catalyst during dehydration, where the epoxy group combines with hydrogen ions in solution to form a hydroxyl group, which in turn combines with the vacant half-bonds, resulting in the detachment of the water molecules and the carbon atoms of the rGO bonded to the sp^2^ bond [22].

**Figure 8:**
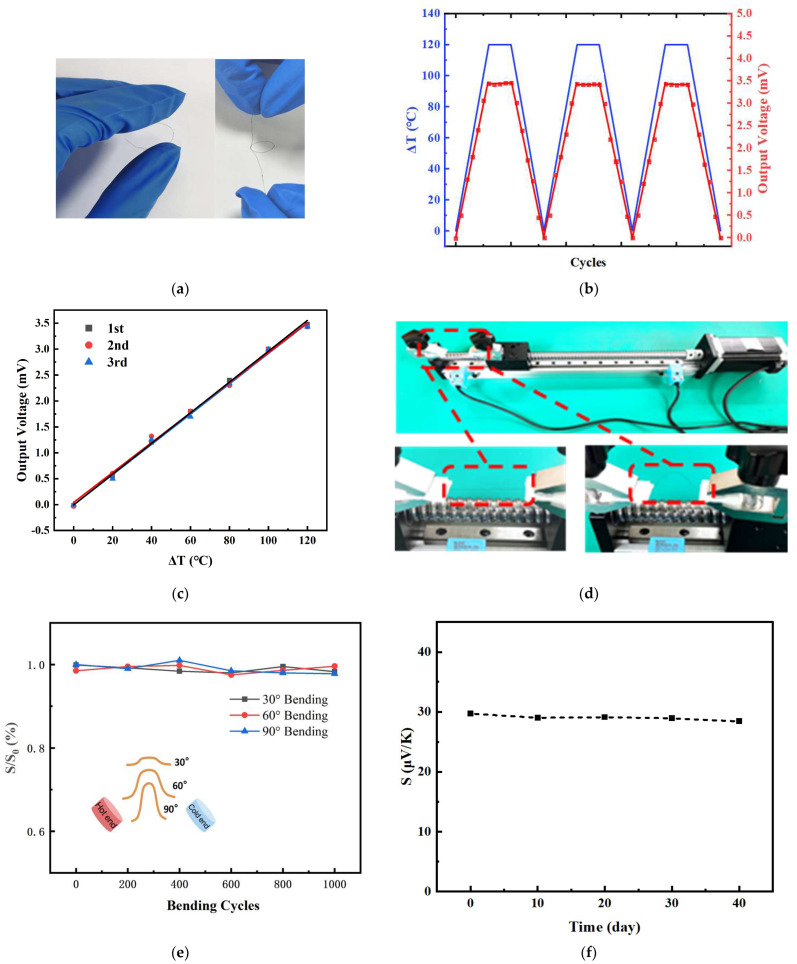
Characterization of GF electrode. (**a**) Bending and knotting diagram of graphene fibers; (**b**) 2 wt%-GF/Pt thermocouple repeatability test; (**c**) Thermoelectric output curves of the thermocouple for three warming processes; (**d**) Diagram of fiber bending and recovery process; (**e**) The sensitivity variation after bending 1000 times in different flexible states; (**f**) Plot of thermocouple sensitivity versus time.

As shown in Figure 8a, the graphene fibers can be bent at any angle and can also be tied into a knot without the fibers breaking, and these results demonstrate the flexibility and torsion resistance of graphene fibers. For thermocouples, their cycle stability and repeatability are important performance parameters. Figure 8b,c showed plots of the output voltage of the thermocouple versus the temperature difference under three repetitions of the test. Fitting the test data over multiple warm-ups reveals that both the temperature difference and the output voltage are linear, and the data from the multiple tests overlap almost exactly, as does the cool-down process. Importantly, the slopes of the plots for multiple warm-ups and multiple cool-downs are almost identical, which indicates that the thermocouples have almost no drift in the test data during multiple warm-ups and cool-downs. The values of goodness of fit obtained by least squares fitting are all greater than 0.99, indicating a good linear relationship. This shows that the thermocouple has good cyclic stability and repeatability.

Figure 8d shows the bending and recovery processes of the fiber. The fibers were clamped on the slide table of the screw guide and were continuously bent and straightened with the rotation of the screw, leading to fixing one end of the fiber and bending back and forth the other end at a speed of 1 mm/s. The fiber was 6 cm long with a radius of curvature of 5 mm. As we can see from Figure 8e, the detecting sensitivities all remain basically unchanged after bending 1000 times in different flexible states (such as 30°, 60°, and 90° bending), suggesting that the thermocouple has the ability to be used for a long time in the bending process.

Figure 8f shows the sensitivity of the fabricated thermocouples over time when left in the air. It can be seen that the sensitivity of the thermocouple encapsulated with PI film shows high stability (>95.66%) over 40 days.

**Figure 9:**
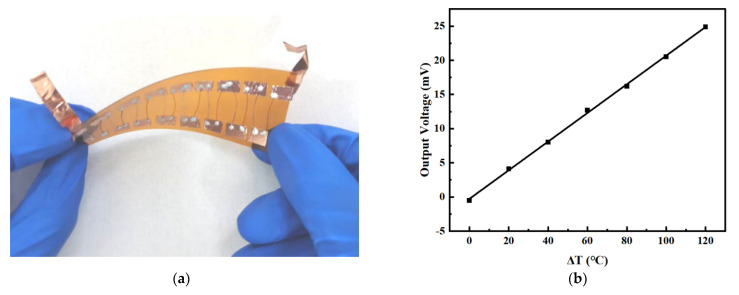
Performance test of multipair thermocouple temperature sensors. (**a**) A temperature sensor having 8 pairs of thermocouples. (**b**) The relationship between temperature difference and output voltage.

Since the length of graphene composite fibers can be prepared as desired, temperature sensors with different pairs of p-n thermocouples can be easily fabricated. Figure 9 illustrates a plot of temperature difference versus output voltage for a temperature sensor with 8 pairs of thermocouples. The results show that the temperature sensor with 8 pairs of thermocouples has a sensitivity of about 200 μV/°C, which produces a substantial increase in sensitivity compared to the temperature sensor composed of 1 pair of thermocouples. Therefore, higher detection sensitivity can be obtained by increasing the number of thermocouple pairs comprising the temperature sensor.

## 4. Conclusions

In this paper, GOFs were successfully fabricated by the microfluidic spinning technique. Vitamin C was employed as a reducing agent to reduce GOFs to GFs. The study focused on examining the impact of different concentrations of vitamin C (ranging from 1 wt% to 4 wt%) on the synthesis of GFs. To analyze the fibers, SEM maps and Raman spectroscopy were used to examine the surface morphology and internal structural defects. The electrical conductivity of 4 wt%-GFs is about 2448 S/m, which is 20,000 times higher compared to the electrical conductivity of pristine GOFs. The 2 wt%-GF and Pt were used to form a pair of thermocouple temperature sensors for testing, which showed good operating characteristics in the low-temperature measurement range with a sensitivity of up to 29.9 μV/°C. Additionally, it can be found that when there was a temperature difference of 120 °C between the cold and hot ends, the maximum thermoelectric output of the sensor reached 3.45 mV. The sensor also demonstrated resistance to bending interference and showed minimal change in sensitivity even after being bent 1000 times. The sensor encapsulated with PI film still stays considerably stable after being left in the air for 40 days. The sensitivity of the temperature sensor, consisting of 8 pairs of thermocouples, can be increased up to 200 μV/°C. With the benefits of small size, light weight, high sensitivity, and good scalability, graphene fiber-based flexible thermocouple temperature sensors are simple to make and environmentally friendly. They are anticipated to reflect human activities and health conditions via real-time monitoring of human body temperature and demonstrate a wide range of application potentials in the fields of e-skin and smart medicine.

## Figures and Tables

**Table 1:** Output fitting data of GF/Pt thermocouples with different vitamin C doping contents.

Sample	V~ΔT/μV	Seebeck Coefficient/μV·°C^−1^	Correlation Coefficient (R^2^)
GOF/Pt *	V = 28.13 + 4.16ΔT	4.16	0.977
1 wt%-GF/Pt	V = −136.92 + 23.04ΔT	23.04	0.995
2 wt%-GF/Pt	V = −23.30 + 29.90ΔT	29.90	0.997
3 wt%-GF/Pt	V = −104.95 + 27.27ΔT	27.27	0.996
4 wt%-GF/Pt	V = −123.52 + 25.57ΔT	25.57	0.996

* It represents the thermocouple with 0 wt% vitamin C added to GF as the anode and Pt as the cathode.

## Data Availability

Data sharing does not apply to this article.
